# Novel Drug Targets for Neurodegenerative Diseases of Elderly People to Develop Effective Therapeutics: A Comprehensive Analysis

**DOI:** 10.1155/adpp/8847508

**Published:** 2025-11-16

**Authors:** Partha Biswas, MD. Hasanur Rahman, Afrida Tabassum, Tanvir Zaman Shoyshob, Maroua Jalouli, Md. Mohaimenul Islam Tareq, Md. Imtiaz, Humayra Afroz Dona, Abdel Halim Harrath, Md Ataur Rahman

**Affiliations:** ^1^ Department of Genetic Engineering and Biotechnology, Jashore University of Science and Technology, Jashore, 7408, Bangladesh, just.edu.bd; ^2^ Department of Biotechnology & Genetic Engineering, Bangabandhu Sheikh Mujibur Rahman Science and Technology University, Gopalganj, 8100, Bangladesh, bsmrstu.edu.bd; ^3^ Department of Genetic Engineering and Biotechnology, Jagannath University, Dhaka, 1100, Bangladesh, jagannathuniversity.org; ^4^ Department of Biochemistry and Molecular Biology, Bangladesh Agricultural University, Mymensingh, 2202, Bangladesh, bau.edu.bd; ^5^ Department of Biology, Imam Mohammad Ibn Saud Islamic University (IMSIU), Riyadh, 11623, Saudi Arabia, imamu.edu.sa; ^6^ Zoology Department, College of Science, King Saud University, Riyadh, 11451, Saudi Arabia, ksu.edu.sa; ^7^ Department of Biotechnology and Genetic Engineering, Islamic University, Kushtia, 7003, Bangladesh, iu.ac.bd

**Keywords:** α-Synuclein, Aβ, blood–brain barrier, genetics, neurodegeneration, therapeutic targets

## Abstract

Neurodegenerative diseases (NDs) represent an increasingly important burden of disease, particularly in the aging population. The etiology of NDs like Alzheimer’s disease (AD), Parkinson’s disease (PD), and Huntington’s disease (HD) is associated with progressive neuronal degeneration and a paucity of effective therapies. Accumulating evidence suggests that common and intersecting genetic and pathological pathways play a critical role in disease onset and progression, revealing new opportunities for target discovery. Here, we review promising therapeutic targets based on the convergence of genetics, molecular pathology, and cellular signaling in neurodegeneration. This narrative will focus on key proteins (amyloid‐beta [Aβ], tau, and α‐synuclein) and enzymes (acetylcholinesterase and asparagine endopeptidase [AEP]), including their pathological significance and therapeutic implications. N‐Methyl‐D‐aspartate receptors (NMDARs) and cholinergic receptor subtypes are highlighted as important regulators of neurotoxicity, synaptic transmission, and inflammation. Emerging advances in genomics, neuroimaging, and drug delivery are poised to advance precision medicine strategies for early diagnosis and intervention. Important challenges remain, including the complexity of the blood–brain barrier (BBB), pathology heterogeneity, and the need for new biomarkers. We propose that a shift from phenotype‐driven diagnoses to mechanistic, genetically informed approaches may improve treatment efficacy. Target identification, validation, and targeted delivery are critical considerations for the success of future therapeutic development. This integrated view will help to inform and improve drug discovery and personalized medicine approaches in the field of neurodegeneration.

## 1. Introduction

Neurodegenerative diseases (NDs) are a category of chronic and progressive disorders marked by a gradual decline of neuronal structure and function, resulting in cognitive, behavioral, and motor deficits [1]. In contrast to static neuronal damage induced by metabolic or toxic factors, NDs lead to an ongoing and selective susceptibility of neuronal populations [2]. Disorders such as Alzheimer’s disease (AD), Parkinson’s disease (PD), and Huntington’s disease (HD) are particularly common among the elderly, presenting considerable medical, social, and economic issues globally [3]. This review focuses on three of the most prevalent and well‐studied NDs—AD, PD, and HD—with an emphasis on emerging drug targets and therapeutic strategies.

AD is the most common form of dementia and is clinically manifested by memory loss, cognitive impairment, and behavioral changes. AD is the predominant cause of dementia in the elderly population. It is marked by extensive neuronal demise and cognitive decline caused by the buildup of amyloid‐beta (Aβ) plaques and tau protein tangles in the brain [4]. These pathogenic characteristics are linked to synaptic failure, oxidative stress, and neuroinflammation, which collectively facilitate the unremitting advancement of AD [5]. Aβ peptides, especially Aβ42, demonstrate significant neurotoxicity owing to their tendency to form soluble oligomers that interfere with neuronal communication and induce death [6]. Tau pathology exacerbates the disease by impeding axonal transport and disrupting microtubules [7]. Recent molecular investigations have demonstrated that the lysosomal cysteine protease asparagine endopeptidase (AEP) cleaves tau and APPs at certain asparagine residues, facilitating tau propagation and Aβ production. Inhibiting AEP has consequently surfaced as a prospective treatment approach [8]. Novel targets such as Aβ aggregation, tau phosphorylation, and neuroinflammatory pathways are currently under investigation to address these unmet therapeutic needs. PDis the second most common NDs. Parkinson’s is characterized clinically by bradykinesia, rigidity, resting tremor, and postural instability [9]. These clinical manifestations result from the gradual degradation of dopaminergic neurons in the substantia nigra and the buildup of alpha‐synuclein‐laden Lewy bodies [10]. α‐Synuclein, a presynaptic protein implicated in synaptic vesicle trafficking and neurotransmitter release, becomes pathogenic due to post‐translational changes including phosphorylation and nitration [11]. These modifications promote oligomerization and aggregation formation, resulting in mitochondrial failure, oxidative stress, and neuroinflammation. Genetic abnormalities in the *SNCA* gene, responsible for encoding α‐synuclein, underscore the significance of protein homeostasis in the pathophysiology of PD [12]. The search for disease‐modifying treatments has broadened to include α‐synuclein aggregation inhibitors, mitochondrial stabilizers, and oxidative stress and neuroinflammation modulators. HD is an inherited neurodegenerative disorder resulting from a CAG trinucleotide repeat expansion in the huntingtin (*HTT*) gene. HD is an autosomal dominant disorder resulting from the increase of CAG trinucleotide repeats in the *HTT* gene, which encodes the huntingtin protein [13]. This growth results in the creation of toxic polyglutamine tracts, leading to neuronal malfunction and death, especially in the striatum and cortex [14]. Individuals with HD generally have a confluence of motor impairments, cognitive abnormalities, and mental manifestations [15]. Although its genetic origin is comprehensively recognized, effective therapeutic strategies are still absent, with most treatments focused on symptom relief. Therapeutic strategies currently under investigation for this disorder include gene silencing, protein misfolding modulators, and homeostatic restoration agents.

The constraints of current therapies highlight the pressing necessity for a more profound comprehension of the fundamental disease mechanisms. Recent advancements in genetics, neuroimaging, and molecular biology have started to elucidate the intricate interaction between genetic predisposition and disease processes [16]. Genome‐wide association studies (GWAS) have revealed many susceptibility genes for AD and PD, highlighting the polygenic characteristics of both conditions [17]. Moreover, advanced imaging techniques like positron emission tomography (PET) and magnetic resonance imaging (MRI) enable the early identification of pathological alterations, including amyloid and tau accumulation in AD and dopaminergic deficiencies in PD [18]. Cerebrospinal fluid biomarkers, such as Aβ42 and phosphorylated tau, are becoming significant in early diagnosis and therapy assessment.

Furthermore, comprehending the intersecting molecular pathways—such as mitochondrial failure, autophagy dysregulation, and neuroinflammation—is essential for pinpointing shared targets that may provide wider therapeutic applicability [19]. Dysfunction in protein clearance pathways is a common characteristic of AD, PD, and HD [20]. Likewise, abnormal activation of glial cells and persistent inflammation facilitate disease advancement in certain neurodegenerative disorders [21]. In the present review, ND in the aged is a global health issue with a major impact on individuals and society. While great strides have been made in our understanding of the molecular and clinical bases of these diseases, targeted therapies remain limited. A multifaceted approach that includes genetic findings, biomarker development, advanced imaging, and innovative drug delivery systems represents the most likely path forward. Continued research on the interplay between genetic and pathogenic processes will be essential for translating these advancements into meaningful clinical therapies.

## 2. Progression of NDs in Elderly People

**Figure 1:**
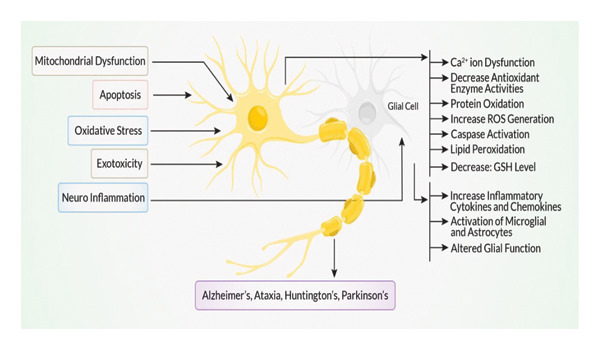
The progression of neurodegenerative diseases in elderly people. Oxidative stress is further aggravated by mitochondrial dysfunction, which not only disturbs energy metabolism but also generates more ROS. Apoptosis that is brought on by mitochondrial mechanisms and exogenous triggers causes neuronal death. The release of excessive glutamate causes excitotoxicity, which further aggravates neuronal injury. Activated glial cells, including microglia and astrocytes, mediate neuroinflammation by increasing the release of proinflammatory cytokines, which results in neuronal damage. These mechanisms interact and amplify the death and injury of neurons, leading to a vicious cycle, eventually contributing to the development of neurodegenerative disorders.

The advancement of neurodegenerative illnesses in older adults is propelled by interrelated molecular pathways that result in neuronal injury and degeneration [22]. Mitochondrial malfunction is pivotal to this process, hindering ATP synthesis and facilitating the overproduction of reactive oxygen species (ROS) [23]. Increased ROS induces oxidative stress, leading to lipid peroxidation, protein oxidation, and a reduction in antioxidant enzyme activity and glutathione (GSH) levels [24]. Simultaneously, apoptotic signaling is initiated via caspase pathways, resulting in programmed neuronal demise [25]. Excitotoxicity, frequently resulting from elevated glutamate levels, aggravates calcium ion (Ca^2+^) dysregulation, hence amplifying neuronal damage (Figure 1). Concurrently, neuroinflammation arises as a significant factor. Activated microglia and astrocytes secrete proinflammatory cytokines and chemokines, sustaining a chronic inflammatory milieu [26]. This modified glial function undermines neuronal support systems and exacerbates neurodegeneration. The interplay of oxidative damage, glial malfunction, and apoptotic loss leads to the gradual decline of neuronal circuits [27]. Ultimately, these molecular disruptions are fundamental to the clinical presentation of many NDs, including AD, PD, HD, and other types of ataxia [28]. Comprehending these pathways establishes a basis for creating targeted therapeutics intended to prevent or reverse neurodegeneration in aging populations.

## 3. Recent Advances in Understanding NDs

NDs are a significant public health problem that affects millions of people all over the world. It’s extremely important for people, families, society, and governments to be affected by these disorders. There have been exciting advances in recent years in understanding the mechanisms underlying these disorders, and they open new diagnostic strategies and therapeutic methods for the treatment of patients. The purpose of this review is to present and update our knowledge about the pathomechanisms of neurodegenerative disorders, focusing on AD, PD, and ALS. Imaging techniques, such as MRI and PET, allow us to observe AD‐related changes in the brain early in the course of the disease and to make an early diagnosis and treatment course [29]. Moreover, GWASs have discovered new genetic risk factors for AD, shedding light on the intricate genetic architecture of the disease [30]. Targeted therapies aimed at reducing Aβ accumulation or tau pathology are currently under investigation, offering potential disease‐modifying strategies for AD [31]. Emerging evidence suggests a role for mitochondrial dysfunction, neuroinflammation, and impaired protein clearance pathways in PD pathogenesis [32]. Additionally, the discovery of genetic abnormalities linked to family variants of PD has shed light on the genesis and pathophysiology of the disease [33]. Novel therapeutic approaches targeting alpha‐synuclein aggregation, mitochondrial function, and neuroinflammation hold promise for slowing disease progression and improving clinical outcomes in PD patients [33]. Recent studies have revealed the involvement of aberrant RNA metabolism, protein aggregation, and glial dysfunction in the pathogenesis of ALS [34]. Advances in gene sequencing technologies have identified numerous genetic mutations linked to ALS, including mutations in the C9orf72, SOD1, and TARDBP genes [35]. In addition, the creation of patient‐derived induced pluripotent stem cells, or iPSCs, has given researchers studying ALS access to a useful platform for drug screening and disease modeling [36]. Emerging therapeutic strategies targeting RNA dysregulation, protein homeostasis, and neuroinflammation offer hope for the development of effective treatments for ALS [37]. By leveraging cutting‐edge technologies and interdisciplinary collaborations, researchers are making significant strides toward the development of precision medicine approaches for neurodegenerative disorders, ultimately aiming to improve patient outcomes and quality of life.

## 4. Key Pathological Proteins and Candidate Genes in NDs

NDs, including AD, PD, and HD, are hallmarked by an accumulation of pathological proteins and specific susceptibility genes. The dysregulation of these molecular determinants induces disease initiation and progression by perturbing neuronal architecture and function. We summarize major pathological proteins and candidate genes in these disorders in Table 1.

**Table 1:** Key pathological proteins and candidate genes in neurodegenerative diseases, their associated mechanisms, and therapeutic approaches.

Sr. no.	Target	Disease	Mechanism	Therapeutic approach	References
1	Amyloid‐β (Aβ)	AD	Formation of extracellular plaques that disrupt synaptic function and lead to neuronal death	Inhibition of aggregation; clearance by immunotherapy	[38, 39]
2	Tau	AD	Hyperphosphorylation resulting in neurofibrillary tangles and microtubule destabilization	Kinase inhibitors; tau aggregation inhibitors	[40, 41]
3	α‐Synuclein	PD	Misfolding and aggregation into Lewy bodies leading to dopaminergic neuron degeneration	Targeting hydrophobic NAC domain; immunotherapy	[42, 43]
4	Huntingtin (*HTT*)	HD	Mutant protein with polyglutamine expansion leading to protein misfolding and neuronal toxicity	Gene silencing (ASOs, RNAi); protein aggregation modulators	[44, 45]
5	BACE1	AD	Beta‐secretase cleaves APP at the β‐site, releasing the Aβ peptide	BACE1 inhibitors	[45, 46]
6	NMDARs	AD, PD	Excitotoxicity caused by excessive glutamate stimulation leads to neuronal death	NMDA receptor antagonists (e.g., memantine)	[47, 48]
7	LRRK2	PD	Mutations lead to abnormal kinase activity and vesicle trafficking defects	LRRK2 kinase inhibitors	[49, 50]
8	PINK1/Parkin	PD	Mitophagy defects impair mitochondrial quality control	Enhancing mitophagy; small‐molecule activators	[51, 52]
9	APOE ε4	AD	Genetic risk factor modulates Aβ clearance and lipid metabolism	Modulators of lipid metabolism; immunotherapy	[53, 54]

### 4.1. α‐Synuclein as a PD

**Figure 2:**
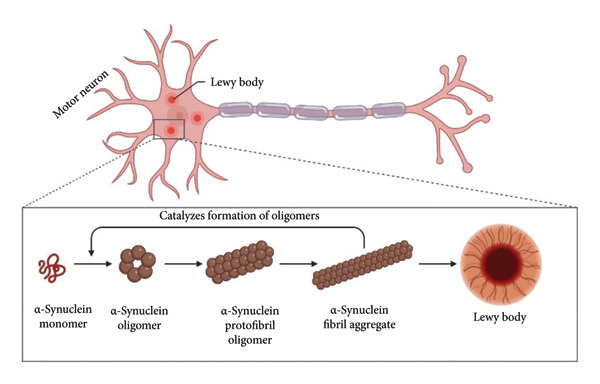
Formation of α‐synuclein aggregation in Parkinson’s disease. Individual α‐synuclein monomers are thought to misfold and form soluble oligomers, which then aggregate to form larger, less soluble protofibril oligomers. As aggregation progresses, these protofibril oligomers transition into fibril aggregates, which are noted for their elongated and filamentous structure. Ultimately, the accumulation of fibrils leads to the formation of Lewy bodies, the characteristic intracellular inclusions that can be observed in the neurons of Parkinson’s disease patients. The figure was adapted and retrieved from Biorender.com on September 02, 2024.

Numerous putative functions of α‐synuclein have been suggested, including its involvement in neuronal survival, binding of fatty acids, trafficking and release of synaptic vesicles, and physiological regulation of certain enzymes, transporters, and neurotransmitter vesicles [55]. PD is categorized as a synucleinopathy and is a progressive neurological disorder with 140 amino acid residues (Figure 2). The α‐synuclein is a tiny soluble cytoplasmic protein [25], which the NG_011851 (*SNCA*) gene encodes [56]. This protein can be detected in the brain’s soluble and membrane‐associated fractions, and it is highly concentrated in presynaptic terminals [57, 58]. Its primary protein domains are the N‐terminal domain (1–60), which has four 11‐amino acid deficient repeats (coding for amphipathic α‐helices) with a retained motif (KTKEGV); the central nonamyloid component (NAC) domain (61–95), which is in charge of its tendency to misfold into amyloid aggregates rich in beta sheets, with three additional KTKEGV repeats and a highly acidic residue‐enriched region; and the C‐terminal domain (96–140), which has several negatively charged residues along with a serine and three tyrosine residues that are phosphorylated, potentially affecting α‐Syn structure, membrane binding, accumulation, and toxicity [57–59].

It has been shown that α‐synuclein plays a role in protecting neurons from different apoptotic factors and in regulating the neuronal apoptotic response. At least 30 proteins have been demonstrated to physically interact with α‐synuclein, which supports its crucial role in cell signaling [58]. Numerous post‐translational modifications (PTMs) of the α‐synuclein protein occur, such as DA modification, nitration, and phosphorylation, all of which seem to encourage oligomerization [30], indicating that these alterations might be involved in the pathophysiology of PD. PD may be significantly influenced by compromised lysosomal clearance systems, in addition to encouraging the buildup of soluble α‐synuclein oligomers [60]. Also, point mutations (A53T, A30P, E46K, G51D, and many others) and multiplications (duplications or triplications) of the *SNCA* gene (which codes for α‐synuclein) have been found in individuals with autosomal dominant familial PD [61]. The wild‐type α‐Syn is present in the healthy brain and central nervous system (CNS) as soluble monomers in its original configuration. It’s possible that native α‐Syn maintains presynaptic function and controls transmitter release. Misfolded α‐Syn oligomers and aggregates have been shown to disturb neuronal homeostasis through a number of pathways, including oxidative and endoplasmic reticulum stress, defects in vesicular trafficking, malfunctions in the autophagy–lysosomal pathway (ALP), and malfunctions in the mitochondria [59]. While fibrils of α‐Syn arranged in β‐sheets are characteristic of LB [24], in vitro studies have shown that oligomer formation, rather than fibrils, is a special characteristic of α‐Syn mutations such as A30P and A53T, which are known to cause early‐onset familial PD [62]. The hydrophobic NAC domain, consisting of 12 amino acids, is the central region of the α‐Syn protein and plays a crucial role in its transition from a soluble monomer to oligomers, protofibrils, and ultimately fibrils that aggregate [57]. The breakdown of α‐synuclein is facilitated by multiple proteolytic systems, such as the ALP and the ubiquitin–proteasome system (UPS) [60, 63, 64]. The UPP disruption caused by aggregation and other forms of autophagy is associated with the gradual accumulation of α‐SYN that is characteristic of PD [65]. As α‐Syn protein plays a crucial role in the development and pathophysiology of PD, it was deemed a promising and obvious treatment target for the disease. The understanding of α‐synuclein’s structure, folding, and function has advanced due to recent structural breakthroughs, indicating that a planned treatment for PD and other synucleinopathies is now more plausible than ever.

### 4.2. Aβ and Tau in AD

**Figure 3:**
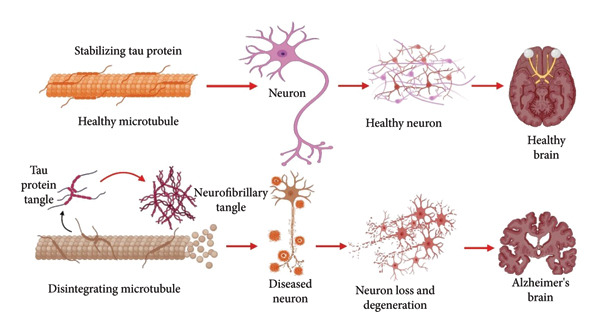
The pathology of Alzheimer’s disease compared to a normal condition: Healthy neurons maintain the microtubules needed to build neurons. Neurofibrillary tangles result from the accumulation of tau protein tangles that cause Alzheimer’s disease, leading to microtubule instability and aggravating neuronal loss and dysfunction. Thus, a substantial decrease in the number of neurons and prominent neuronal degeneration ultimately leads to extensive neurodegeneration. The typical pathological alterations of the disease lead to progressive cognitive impairment and memory loss caused by the disease. The figure was modified and obtained from Biorender.com on September 02, 2024.

β‐ and γ‐Secretases sequentially digest the APP to produce amyloid peptides (Aβ), which are 39–43 amino acid peptides that make up amyloid plaques [66]. Proteolytic degradation, cellular clearance via lysosomal degradation in brain cells like microglia, astrocytes, and neurons, and cerebrovascular system‐accelerated clearance are the three main ways that Aβ is removed from the brain [67]. AD patients’ brain tissue contains higher quantities of Aβ peptides [68], and among these peptides, Aβ42 is considered the most cytotoxic to cells [69]. The Aβ peptide exists in a variety of forms, such as insoluble fibrillar aggregates, soluble oligomers, intermediate protofibrils, and soluble monomers [70]. The severity of AD is strongly associated with the concentration of soluble Aβ oligomers among various Aβ species. These oligomers have the potential to induce neuronal damage, leading to the development of dementia [71]. Physiological Aβ concentrations are essential for the occurrence of proper synaptic plasticity and memory. However, an atypical level of Aβ concentration is strongly linked to alterations in behavior and impaired cognitive performance. When healthy rats are given Aβ oligomers, their spatial memory might be significantly impaired. Levels of certain Aβ oligomers in transgenic mice are highly correlated with spatial memory impairments [72]. Aβ peptide has been shown in numerous investigations to be toxic to neurons in lab settings, and its toxicity is linked to its fibrillar state and β‐sheet structure in primary neurons or neuronal cell lines [73]. Most of the biochemical details about how extracellular Aβ peptide harms neurons remain unclear. It is reported that Aβ interacts with components of cell membranes, leading to further damage to neurons or making them more susceptible to harm, which is one of the primary ways that Aβ causes neurotoxicity (Figure 3) [74].

### 4.3. AEP

AEP is a cysteine protease located in endolysosomes that cleaves its substrates specifically at asparagine residues, and its activity is dependent on the pH of its environment. Different latest investigations have discovered that it has delta‐secretase activity and is associated with several NDs like stroke and AD. Increasing data suggests that inhibiting AEP has positive effects in treating these severe disorders [75]. Recent data makes it abundantly evident that AEP cleaves its substrate, such as tau, SET, and APP, and that it is essential to the death of neurons in stroke and other neurodegenerative disorders [75]. While AEP is engaged in numerous physiological and pathological processes, such as immunity and the course of cancer, our research was the first to clarify its biological function in the nervous system in 2008 [76]. We showed that when brain ischemia and epileptic seizures cause brain acidosis, AEP is triggered. An inhibitor of DNase called SET is cut by the active AEP, which also damages brain DNA. A prosurvival protein found in the nucleus and linked to the plasma membrane called PIKE‐L prevents AEP from cleaving SEP [76–78]. Thus, AEP may be one of the main proteinases that is triggered by acidosis and causes neuronal damage during ischemia or neuro‐excitotoxicity. Most recently, we discovered significant quantities of AEP protein in the brain, where it demonstrates the novel delta‐secretase function by age‐dependently cleaving tau and APP. Moreover, compared to healthy people, patients with AD have higher levels of AEP activity in their brain tissues, and this higher activity contributes to the initiation and development of AD [79, 80]. AEP has been found to have a significant impact on axonal regrowth following spinal cord injury in zebrafish, which is consistent with its involvement in neurodegenerative disorders. Throughout the axon regrowth/sprouting phase, neurons of regenerative nuclei expressed more AEP. Suppression of AEP expression hindered the process of axonal regeneration and the restoration of locomotor function [81].

### 4.4. N‐Methyl‐D‐Aspartate Receptors (NMDARs)

NMDARs are a specialized type of ionotropic glutamate receptors that are largely located in the CNS. They are named after their ability to bind specifically to the synthetic chemical NMDA (N‐methyl‐D‐aspartate) [82, 83]. NMDARs consist of two NR1 and two NR2 subunits, which combine to form tetramers. Both glycine and d‐serine can activate the glycine binding site on NR1; however, d‐serine is more effective, especially when considering the composition of the NMDAR subunits. Furthermore, glial cells actively release d‐serine, which suggests that they have a significant role in regulating multiple NMDAR‐dependent functions. These functions include synaptic transmission, synaptic plasticity, rhythmic activity, activation of second messenger pathways, gene expression, and pathophysiological events like excitotoxicity as well as neurodegeneration [82–84]. Various combinations of NMDAR1 (NR1), NMDAR2 (NR2), and NMDAR3 (NR3) subunits produce the macromolecular structures known as NMDA receptors (NDMARs). This molecule is structured along a twofold axis that passes through its three domains: the transmembrane domain (TMD), the ligand‐binding domain (LBD), and the amino‐terminal domain (ATD). The NMDA receptors, which allow the passage of Ca^++^ ions, are structured as a dimer consisting of GluN1–GluN2B heterodimers [82, 85]. The complete activation of NMDA receptors is controlled by both changes in voltage and the binding of certain molecules [86]. The NMDA receptor ion channels exclusively activate when the postsynaptic membrane undergoes depolarization and when the neurotransmitters glutamate and glycine are bound to them. The opening of the channel is prevented by a blockage of Mg2^+^ that is dependent on voltage when the membrane is not depolarized. Depolarization occurs when glutamate binds to AMPA receptors, which enables the entry of Na^+^ and K^+^ ions into the cell. This reduces the negative charge inside the neuron. Binding glutamate and glycine to NMDA receptors results in the opening of ion channels that allow the passage of Ca2^+^ ions. The entry of Ca2^+^ ions into the cell initiates the process of adding phosphate groups to AMPA receptors, which enhances their sensitivity to glutamate and leads to an increase in their abundance within the cell membrane. This maintains membrane depolarization and sustains action potential generation [83, 86, 87]. Excessive stimulation of NMDA receptors, resulting in an abnormally high entry of calcium ions, can lead to excitotoxicity, a process implicated in some neurodegenerative conditions. For synaptic plasticity and neuron survival, excitatory glutamatergic neurotransmission via the NMDAR is essential; however, excessive NMDAR activity results in excitotoxicity and encourages cell death, which may be a contributing factor to the neurodegenerative processes involved in AD [88]. Several research studies have suggested that the specific results of NMDAR‐mediated reactions are caused by localized receptor activity, which then triggers other signaling pathways.

### 4.5. Acetylcholinesterase (AChE)

AChE is well recognized as a primary focus of neurodegenerative illnesses due to several studies that have established a connection between the enzyme and the gradual deterioration of the nervous system’s structure and function. The synaptic enzyme AChE quickly breaks down the neurotransmitter acetylcholine (ACh) in cholinergic synapses in the peripheral and CNS, thus ending neurotransmission [89]. AChE influences apoptosis, oxidative stress, the inflammatory response, and the aggregation of pathogenic proteins, all of which are significant aspects of the pathophysiology of neurodegenerative disorders [90]. The pace at which AChE functions is remarkably quick, similar to that of a diffusion‐controlled process [91]. After solving the crystal structure of *Torpedo californica* AChE [92], the three‐dimensional structure of AChE has played a crucial role in comprehending its remarkable catalytic efficiency, facilitating rational drug design, and advancing therapeutic strategies for neurodegenerative disorders. Additionally, knowledge of the structural details of the ACh‐binding pocket in AChE can aid in comprehending the molecular mechanisms involved in the recognition of ACh by other ACh‐binding proteins such as the muscarinic and nicotinic ACh receptors [91]. The AChE molecule has an ellipsoidal shape with dimensions of approximately 45 × 60 × 65 Å. It consists of 537 amino acids, and the monomer of this enzyme is a protein with a core structure consisting of a mixed β sheet composed of 12 strands and 14 α‐helices. AChE has multiple binding sites, including the catalytic site (CAT), acyl pocket, oxyanion hole, anionic site, and peripheral anionic site (PAS). The active site of the enzyme is located at the bottom of a deep gorge, primarily surrounded by aromatic residues, in a manner that is surprising. The foundation and inner surface of the gorge are composed of remnant deposits of Asn‐66 and Ile‐444. Additionally, it contains residues such as Asp‐285, Glu‐273, Asp‐72, Tyr‐334, and Glu‐199 [93, 94]. The hydrolysis process achieved through a Glu327–His440–Ser200 catalytic triad, the same as other serine hydrolases, and a three‐pronged oxyanion hole works on stabilizing the transition state, as seen in other serine hydrolases [93, 95]. The anionic site comprises Trp‐86, Trp‐84, and Phe‐330 residues, which influence substrate selectivity and produce a flexible “glycine loop” substrate complex [95]. The enzyme’s loop, which is next to S203, interacts with noncovalent AChE ligands. The presence of Trp‐286 and Trp‐86 is crucial for the inhibition caused by PAS ligands, as they contribute to the flexibility of the active site [96]. PAS can function as either a primary binding site or influence the removal of cations and the release of products [97]. The crystal structure of recombinant human AChE in its apo‐state closely resembles that of the Torpedo enzyme in terms of overall features. However, the crystal packing in this structure is unique and includes a novel peptide sequence that blocks access to the active‐site gorge [91]. AChE can exist in three different forms: monomer, dimer, or tetramer. However, the tetrameric form (G4) is more prevalent in the brain, whereas the dimeric form (G2) is mostly found on erythrocytes [94]. AChE inhibitors or nicotinic ACh receptor agonists are frequently used to treat these diseases.

### 4.6. Muscarinic and Nicotinic ACh Receptors

Muscarinic and nicotinic ACh receptors are two of the major classes of cholinergic receptors based on their activating ligands [98]. Nicotinic receptors are activated by the agonist nicotine, while muscarinic receptors (mAChRs) are activated by muscarine. The cholinergic system has a crucial role in cognitive function [99], and ACh, an endogenous neurotransmitter, affects both muscarinic and nicotinic cholinergic receptors, making the functions of both receptor systems in cognitive activities unclear [98]. This is significant since it might impact cholinergic treatment for cognitive problems. The two receptors function differently, with one acting as an ionotropic ligand‐gated receptor and the other as a G‐protein coupled receptor. Nicotinic receptors function within the CNS and at the neuromuscular junctions. mAChRs function in both the peripheral and CNSs, facilitating communication with visceral organs such as the heart, smooth muscles, and glands [98]. Both receptors play a role in attentional processes and have an indirect impact on learning and memory. A research study conducted in 1999 illustrated the distinct functions of the two types of cholinoceptors in the processing of information [100]. mAChRs are categorized into five primary subtypes: M1, M2, M3, M4, and M5 [101]. While each subtype is expressed by distinct genes and distributed in various tissue types, all subtypes occur inside the CNS [98]. The widespread distribution of receptor subtypes across various glands and tissues serves to regulate the parasympathetic division of the autonomic nervous system, hence ensuring internal homeostasis.

## 5. Effective Drug Targets for the Treatment of NDs

**Figure 4:**
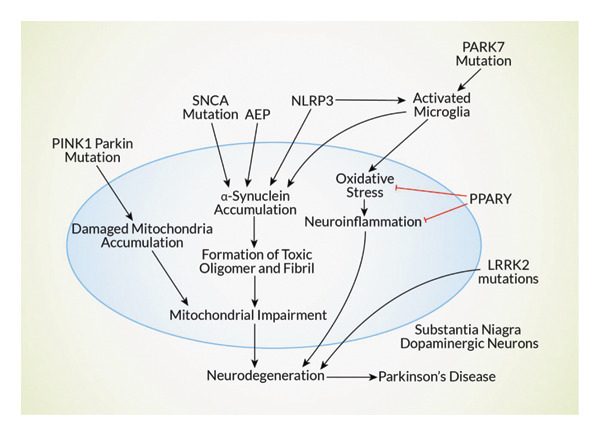
Mechanistic representation of potential drug targets for Parkinson’s disease treatment. Mutations in Parkin and PINK1 have also been associated with mitochondrial dysfunction. Parkin and PINK1 have roles in mitochondrial quality control, and loss of either can result in mitochondrial damage. DJ‐1 PARK7 Mutations in PARK7 (DJ‐1) have also been associated with mitochondrial dysfunction. DJ‐1 guards cells against oxidative stress and mitochondrial dysfunction. Mutations in the gene that prevent these protective activities can impair mitochondrial function. LRRK2 LRRK2 mutations also have effects on mitochondrial dynamics and function. LRRK2 is involved in the maintenance of mitochondrial homeostasis, as well as several other cellular processes. Mutations can lead to changes in these pathways, resulting in mitochondrial dysfunction. Alpha‐synuclein accumulation, especially for mutant forms of alpha‐synuclein, is also known to be damaging to mitochondria. Alpha‐synuclein may have the ability to impair mitochondrial function, reduce energy production, and increase oxidative stress.

Numerous targets for neurodegenerative medications were reported by several investigations. In AD treatment, amyloid is a critical target for the drugs. AD is eventually brought on by inflammation, oxidative stress, and synaptic dysfunction brought on by the Aβ peptides generated from amyloid proteins [102]. Since β‐secretase enzymes cause the development of amyloid proteins, they are regarded as a powerful target. According to reports, BACE1 (β‐secretase/memapsin 2) is a potential therapeutic target because its deletion results in reduced generation of Aβ [102, 103]. Phosphorylation‐induced changes to tau protein result in brain cell death. Studies reported that treatment for NDs can benefit from tau removal [104]. Apolipoprotein E’s involvement in amyloid trafficking has led to reports of it being a useful target [102]. Targeting AChE in AD patients reduces the cholinergic system, which is linked to cognitive decline [105, 106] (Figure 4).

Activating the M1 subtype of mAChRs can improve cognitive function in AD by lowering tau phosphorylation and upregulating α‐APP [107, 108]. It can also cause hypocholinergic effects, which occur because of Aβ production. Oxidative stress is produced by increased monoamine oxidase‐B (MAO‐B) activity, and MAO‐B has been identified as a therapeutic target for the treatment of AD and PD [109, 110]. The death of substantia nigra cells, which release dopamine, is the cause of PD. Numerous investigations have revealed that obstructions in the dopamine pathway are caused by aberrant expression of the *SNCA*, PARK2, PINK1, DJ1, LRRK2, and ATP13A2 genes [104]. It was successful in suppressing leucine‐rich repeat kinase 2 (LRRK2) by specifically targeting LRRK2 kinase [111]. The *SNCA* mutation in PD leads to the increased aggregation of α‐synuclein protein [112]. Treating PD and related disorders could be achieved by effectively targeting α‐synuclein [113]. PTEN‐induced kinase 1 (PINK1) degrades dysfunctional mitochondria under stressful circumstances. PINK1 initiates mitophagy by recruiting Parkin (PARK2, *PARK6*). Mutations in the PINK1 and Parkin result in neurodegeneration by starting the buildup of damaged mitochondria, which eventually causes PD [114].

**Figure 5:**
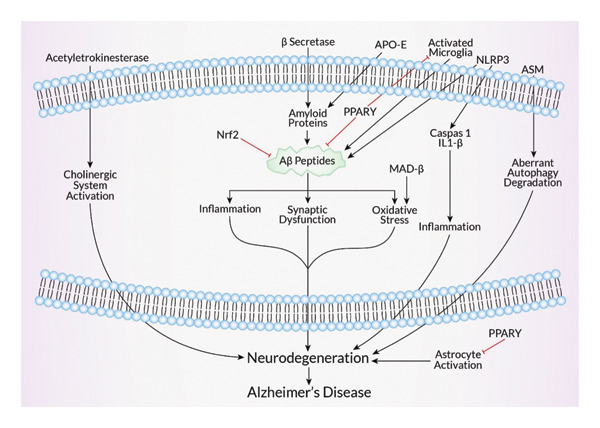
Mechanistic representation of potential drug targets for Alzheimer’s disease treatment. Activated caspase‐1 and IL‐1 beta mediate an inflammatory response that is crucial to activate astrocytes and neuroinflammation. As caspase‐1 processes proinflammatory cytokines such as IL‐1 beta, it may activate astrocytes. Activated astrocytes may increase proinflammatory mediators and exacerbate neuroinflammation and/or lead to neurodegeneration. ASM plays a role in neurodegeneration of AD in a variety of ways. Ceramide levels increase with ASM activity. Ceramide accumulation is associated with neuronal death, synapse dysfunction, and cognitive impairment. Ceramide has been shown to cause neuronal death and synapse dysfunction. ASM activity and ceramide levels are positively correlated in models of AD. Ceramide can further augment AD pathogenesis by activating inflammatory pathways. Ceramide is involved in the development of AD by promoting Aβ aggregation and amyloid plaque formation. In AD, cellular homeostasis is dependent on dysregulation of ASM impairing lysosomal breakdown.

The aberrant function of another neuroprotective DJ‐1 encoding gene, PARK7, is linked to PD. PD can be slowed down in its course by focusing on these genes [115]. Dysfunctional NMDARs are related to the progression of various NDs. NMDAR‐targeting therapeutics produced encouraging outcomes [116]. Mutations in the ATP13A2 gene may cause lysosomal function to be disrupted, which impedes the elimination of cellular waste and ultimately results in neurological disease [117]. Since AEP facilitates the formation of α‐synuclein protein aggregates, targeting AEP is a viable treatment strategy for NDs [75, 118]. According to studies, one excellent alternative for treating a variety of neurodegenerative disorders would be to target astrocytes [119, 120]. Given that PD is linked to dysregulated microRNA, miRNA has been suggested as a potential target for medication [121, 122]. Nuclear factor E2‐related factor 2 (Nrf2) has been identified as a possible target for treating NDs. In many neurological illnesses, neuroinflammation and impaired mitochondrial function are common hallmarks. Free radical scavenging and the regulation of numerous essential mitochondrial enzymes are two ways that Nrf2 is known to protect mitochondria [123]. According to one study, transgenic mice with Nrf2 deletion have higher levels of the AD marker (Aβ and phosphorylated tau protein) [124, 125] (Figure 5). The increased level of acid sphingomyelinase (ASM) is correlated with AD and PD progression, making it a therapeutic target. In AD, ASM promotes aberrant autophagic degradation. ASM in PD is responsible for raising ceramide levels in plasma, which is associated with PD pathogenesis [126]. Microgliosis induces neuronal death through neuroinflammation, and oxidative stress contributes to NDs. Microglia contribute to neuronal damage in AD and PD by producing Aβ and α‐synuclein, respectively. Drugs targeting microglia would be a great option for neuroprotection [127]. Peroxisome proliferator‐activated receptors‐γ (PPARγ) are reported as a drug target for neurological disease. Activating PPARγ reduced oxidative stress and neuroinflammation in PD rat models, as well as Aβ deposition, COX2 expression, and microglia astrocyte activation in AD mice models [128]. The activation of the nucleotide‐binding oligomerization domain‐like receptor pyrin‐containing 3 (NLRP3) inflammasome accelerates the neurological disorder’s progression. Studies showed that in NLRP3‐defective AD mouse models, Aβ clearance is increased and caspase‐1 and IL1‐β activation are reduced. NLRP3 is involved in PD progression by activating both α‐synuclein and microglia [129].

## 6. Current Therapeutic Challenges and Limitations of NDs of Elderly People

Despite extensive research over several decades, neurological diseases in the elderly—such as AD, PD, and HD—remain significant therapeutic obstacles. These diseases are marked by a progressive and irreversible loss of neurons and synaptic function; however, there are no therapeutic treatments capable of halting or reversing this neuronal degeneration. The intricacy of their pathophysiology, along with the inaccessibility of cerebral tissue and the constraints of existing models, obstructs the advancement of viable treatments.

A major problem resides in the mechanistic variability of these disorders. Although hallmark characteristics including Aβ plaques, tau tangles, alpha‐synuclein aggregates, and polyglutamine expansions are well reported [130], they constitute merely a segment of the disease spectrum. NDs encompass various interconnected pathways, such as mitochondrial malfunction, oxidative stress, proteostasis imbalance, excitotoxicity, and neuroinflammation [131]. These pathways may vary considerably among patients, even those with identical clinical diagnoses, leading to treatment variability and therapy resistance. A significant constraint is the dependence on clinical characteristics for the definition and treatment of many disorders. Neurodegenerative disorders have conventionally been categorized and treated according to manifest symptoms, such as memory deterioration or motor dysfunction [132]. Nonetheless, these clinical characteristics frequently manifest following significant neurodegeneration, hence diminishing the opportunity for effective management. Moreover, analogous symptoms may emerge from different molecular diseases, confounding diagnosis and resulting in possible misdiagnosis.

The advent of nonclinical phenotypic methodologies, like molecular and genetic profiling, presents a viable option to address this constraint. GWAS, transcriptome analysis, and biomarker identification have uncovered varied genetic fingerprints even within a singular diagnostic category [133]. Recent data in AD research indicates that certain patient subsets display predominant tau pathology without substantial amyloid accumulation [134], whilst others exhibit inflammatory or vascular‐driven alterations. These disparities are frequently undetectable in conventional clinical evaluations, highlighting the necessity for a classification system grounded in mechanistic understanding.

Nonetheless, the execution of these nonclinical strategies introduces more challenges. Initially, the identification of dependable and accessible biomarkers that correlate with fundamental molecular dysfunction presents a significant challenge. Numerous prospective biomarkers, like phosphorylated tau and Aβ42, necessitate invasive techniques such as lumbar puncture [135], hence constraining their regular application in clinical settings. Secondly, although genetic variations linked to illness risk—such as APOE4 in AD or LRRK2 in PD—have been identified, the translation of these discoveries into viable treatment targets has progressed slowly due to an inadequate comprehension of their functional implications. Moreover, the blood–brain barrier (BBB) constitutes a significant obstacle to efficient drug delivery [136]. Most therapeutic agents are unable to traverse the BBB at adequate concentrations to provide significant effects. Strategies like nanoparticle‐based delivery systems, receptor‐mediated transcytosis, and transitory BBB disruption are now under investigation; however, they remain in preliminary experimental stages.

## 7. Conclusion

NDs in the elderly, such as AD, PD, and HD, present considerable clinical and research challenges owing to their complex and multifactorial characteristics. These disorders are characterized by the progressive dysfunction and death of neurons, influenced by mechanisms including protein aggregation, oxidative stress, mitochondrial dysfunction, neuroinflammation, and impaired proteostasis. Despite extensive research over several decades, existing therapeutic strategies primarily provide palliative care, concentrating on symptom management rather than disease modification or reversal. Recent developments in genomics, transcriptomics, and proteomics have uncovered significant heterogeneity in disease pathogenesis that traditional clinical phenotypes fail to capture. The paradigm is shifting toward nonclinical phenotype‐based approaches that prioritize molecular signatures, biomarker discovery, and individualized therapeutic targeting. However, considerable obstacles persist. The advancement of effective disease‐modifying therapies is constrained by limited access to human brain tissue, inadequate translation from animal models, and the restricted ability of therapeutics to penetrate the BBB. Furthermore, although genetic and protein‐based biomarkers show potential for diagnosis, their clinical use is limited by issues of invasiveness and variability. Future efforts should focus on integrative, multi‐omics strategies that analyze the interaction between genetic risk factors and neuropathological processes. Innovative approaches for early detection, individualized treatment, and precise drug delivery will be crucial. Highlighting mechanistic pathways and delineating molecular subtypes of disease may transform the management of neurodegenerative disorders and enhance the quality of life for older adults. Connecting clinical symptoms with underlying molecular pathology is essential for developing effective therapies for these debilitating disorders.

## Conflicts of Interest

The authors declare no conflicts of interest.

## Author Contributions

Research idea development and conceptualization by Partha Biswas and Md Ataur Rahman; writing and main draft preparation by Partha Biswas, Afrida Tabassum, Tanvir Zaman Shoyshob, Md. Mohaimenul Islam Tareq, Humayra Afroz Dona, and Md Ataur Rahman; figures drawing by MD. Hasanur Rahman; review and editing by Maroua Jalouli, Abdel Halim Harrath, and Md Ataur Rahman. visualization and supervision by Partha Biswas and Md Ataur Rahman. All authors have revised and agreed to the published version of the manuscript.

## Funding

No funding was received for this manuscript.

## Data Availability

The data that support the findings of this study are available from the corresponding author upon reasonable request.
